# SBO ACTION: conservative Small Bowel Obstruction management in the Absence of standard ConTrast agents ON outcomes

**DOI:** 10.1093/bjs/znaf104

**Published:** 2025-06-24

**Authors:** SBO ACTION Collaborative, Josephine Walshaw, Daniel Ashmore, Adam Peckham-Cooper, Matthew J Lee, Raimundas Lunevicius, Adam Daniel Gerrard, Jay Roe Tan, Ross Nieuwoudt, Jacob Mewse, Alice Luesley, Nicola Eardley, Rachael Clifford, Lucia Sepesiova, Tim Wilson, Thomas Hall, Shoieb Mridha, Jessie Blackburn, Ajay Belgaumkar, Khaldoun Fozo, Sukanya Thavanesan, Oroog Ali, Alex McCulla, Ross Lilley, Corin Lathan, Rory Austin, Chathura Munasinghe, Milad Tavakoli, Ademola Adeyeye, Amyn Haji, Ayinke Dosu, Kasthoory Kandiah, Panagiotis Kapsampelis, Ioannis Gerogiannis, Georgios Bointas, Negar Ghaffari, Shirley Chan, Keshav Jindal, Danny Lamdin, Devika Nair, Sita Kotecha, Ala Saab, Alexander Wilkins, Annabelle Williams, Naomi Warner, Anamaria Schipor, Liam Martin, Ashuvini Mehendran, Marianne Hollyman, Ahmed Abdal Rahim, Mike Richardt, John Wayman, Hannah Dunlop, Ning Xuan Ho, Olivia Cory, Michael Okocha, Alasdair Ralston, Luke Williams, Michael El-Boghdady, Hussayn Shinwari, Edward J Nevins, Kohei Yamada, Aya Musbahi, Tejinderjit Athwal, Alex Carney, Daisy Evans, Tomasz Galus, Victoria Gregory, Sam Jacobs, Nicholas Newton, Andrew Daley

## Introduction

Bowel obstruction is a common condition, accounting for 12–16% of acute surgical admissions^1^. Small bowel obstruction (SBO) is the most frequent site, comprising half of all emergency laparotomies performed in England and Wales between 2019 and 2020^2^. The leading cause of SBO is intra-abdominal adhesions, with adhesional small bowel obstruction (aSBO) accounting for approximately 60% of cases^3^. Optimal management of aSBO remains a subject of ongoing debate.

Non-operative management (NOM) for aSBO can be successful in over 70% of patients, with lower rates of short-term morbidity and mortality compared to early surgical intervention^3^. However, it is crucial to promptly identify patients unsuitable for NOM and ensure timely surgical intervention when necessary, as delays exceeding 72 h significantly increase perioperative morbidity^4^. CT imaging is important in decision-making, helping to detect bowel compromise, which usually contraindicates NOM^5^.

Patients without clinical or radiological evidence of bowel compromise can safely undergo initial NOM, which includes gastrointestinal (GI) decompression with a nasogastric tube, fluid and electrolyte replacement, and early administration of water-soluble contrast agents (WSCA). WSCAs have a hyperosmolar effect on the bowel, which is thought to promote the resolution of aSBO by drawing water into the intestinal lumen. Although direct animal studies evaluating this effect are lacking, clinical observations and prior studies suggest that WSCAs may have a therapeutic role in addition to their established function as a diagnostic stratification tool. Although opinions on their administration approach and therapeutic effects vary^6^, they play a key role in assessing the likelihood of success for NOM^5^. Gastrografin (diatrizoate) is the most common WSCA used for aSBO. Although alternative contrast agents such as Omnipaque (iohexol) and Urografin (amidotrizoate) are widely used diagnostically and have excellent safety profiles, there is limited evidence supporting their use as treatment options for aSBO.

A shortage of Gastrografin due to supply chain challenges^7,8^ raised concerns about the impact on treatment and outcomes for NOM patients. This study aimed to assess the management and outcomes of patients presenting with aSBO in the context of a shortage of Gastrografin.

## Methods

Conservative Small Bowel Obstruction management in the Absence of standard ConTrast agents ON outcomes (SBO ACTION) was a national, multicentre, prospective cohort study conducted in the UK from 1 April to 1 July 2024. This study is reported in accordance with the STROBE statement^9^.

As SBO ACTION was a combined audit and service evaluation, ethical approval was not required. Each participating site secured local audit and Caldicott Guardian permissions to participate and were not permitted to collect data without confirmation of approvals.

### Study setting

Eligible sites included any National Health Service (NHS) hospital in the UK providing emergency surgical services. Each participating hospital had a local principal investigator, responsible for coordinating and organizing local teams, and up to three local collaborators. Roles are detailed under the heading ‘SBO ACTION Collaborative’, in line with the guidelines for authorship in collaborative research^10^. To ensure consistency in data collection and management, all participating centres received standardized training via an online presentation delivered by the study steering committee. However, contrast administration protocols were not strictly standardized across sites, as this study aimed to capture real-world variation in practice.

### Study population

Consecutive adult patients (aged ≥16 years) referred to emergency surgical services with a working diagnosis of SBO were screened for inclusion. Patients were included if they had a positive CT confirming aSBO with the intention to manage conservatively for at least 24 h, as determined by a specialist surgeon (ST3+).

Cases with prior abdominal surgery in the same admission, non-adhesional SBO, pregnant women, those <16 years old, and those with a hospital length of stay <24 h were excluded.

### Data collection

Patients were prospectively identified over a three-month data collection period between 1 April and 1 July 2024. Data were collected using prespecified Case Report Forms (Supplementary Material  *S1*) and entered into a secure online Research Electronic Data Capture system^11^, hosted by the Leeds Teaching Hospitals NHS Trust. Data collected included:

- Admission and demographic data: timing of admission, department of first presentation, prior admissions with aSBO, Clinical Frailty Score (CFS), Charlson Co-morbidity Index (CCI).
- Baseline physiology: renal function, venous lactate.
- Diagnostic tests: use of plain abdominal radiography and CT abdomen.
- Contrast use: type and volume of contrast, timing of radiography, repeated challenges.
- Operative management: approach, bowel ischaemia assessment.
- Care episode data: length of stay, unplanned intensive care use.
- Clinical outcomes: deaths, infective complications, postoperative complications, unplanned readmission within 30 days.

All data were routinely collected and captured from clinical records. The Rockwood CFS was used to assess patient frailty, categorizing individuals on a 9-point scale based on mobility, co-morbidities, and functional independence^12^. Scores range from 1 (very fit) to 9 (terminally ill), with higher scores indicating greater frailty and vulnerability to poor outcomes. The CCI was used to quantify co-morbidity burden^13^. It assigns weighted scores to 17 predefined conditions, with higher scores indicating greater co-morbidity and risk of death. The total score was calculated by summing individual condition weights.

### Outcomes

The primary outcome measure was the success rate of NOM, defined as discharge without requiring surgery and survival. Secondary outcomes included rate of progression to surgery, incidence of bowel ischaemia, and in-hospital mortality. Definitions of recorded outcomes are available in Supplementary Material  *S1*. GI recovery (GI-2) was defined as the time to passage of flatus and tolerance of solid food^14^. Time to contrast administration, time to surgery, time to GI-2, and hospital length of stay were recorded in days. Failure of NOM was defined as the occurrence of surgery, in-hospital death, or both.

### Statistical analysis

All analyses were performed in R version 4.4.2 (R Foundation for Statistical Computing, Vienna, Austria). Descriptive statistics were used to summarize quantitative outcomes. Categorical variables are presented as frequencies and percentages, and continuous variables as medians with interquartile ranges. The sensitivity and specificity of contrast in the colon was calculated for those patients undergoing active NOM (that is not converted to palliation). Groups were compared using the *t*-test or Wilcoxon rank-sum as appropriate. Significance was set at *P* ≤ 0.05 *a priori.* Correction for multiple testing was conducted with a Bonferroni correction, and adjusted *P* (q values) also calculated.

A binomial regression model was developed using plausible clinical data to explore the effect of contrast use on the need for surgery. Initial models were developed using one explanatory variable per 10 events. Models were refined to avoid colinear variables, and optimize Akaike Information Criterion (AIC), while adjusting for key baseline clinical factors and investigations such as venous lactate. Key predictors (CFS, CCI, previous aSBO) were chosen based on their association with surgical decision-making in SBO management. The CFS and CCI were included in the regression model as continuous variables, rather than categorical, to preserve granularity. Although interaction terms were considered, they were not included to preserve model stability and interpretability given the sample size constraints.

## Results

**Fig. 1:**
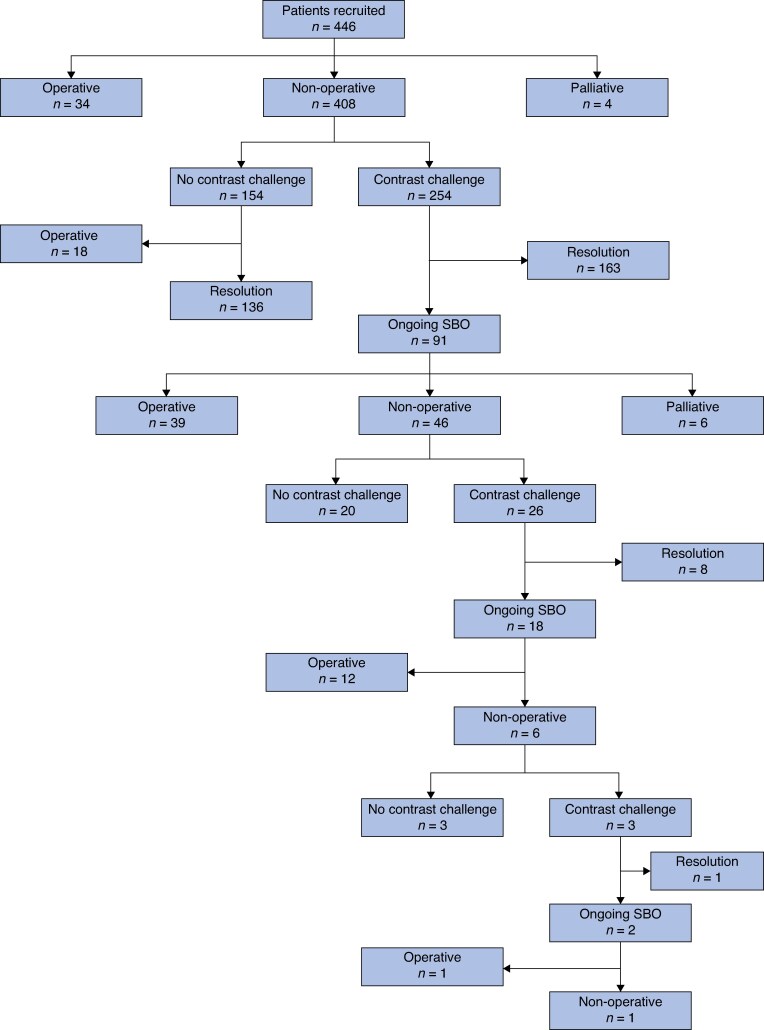
Patient flowchart SBO, small bowel obstruction

Nineteen acute UK hospital trusts identified a total of 446 patients. Of these, 408 (91.5%) underwent an initial trial of NOM (*Fig. 1*). Among the 38 patients (8.5%) who did not have an initial trial of NOM, 34 (7.6%) had a decision for operative management made within 24 h, and 4 patients (0.9%) were palliated; these patients were not included in further analysis.

### Admission data

The median age of patients undergoing NOM (*n* = 408) was 70 years (i.q.r. 58–81), with an approximately even split of male to female patients (180:228). The median CCI was 3 (i.q.r. 2–5). The CFS score was 1–3 in 231 patients (62.6%), 110 (29.8%) showed moderate frailty (scores 4–6), and 28 (7.6%) were very frail (scores 7–9). Of the 408, 150 (36.8%) had undergone previous management of aSBO. Acue kidney injury (AKI) was present in 75 patients (18.4%). Ninety-four patients (23.0%) underwent plain abdominal X-ray (AXR) as part of initial imaging, prior to a diagnostic CT. The demographics of all NOM patients are presented in *Table 1*.

**Table 1:** Demographics of non-operatively managed patients

Characteristic	No contrast (*n* = 154)*	Contrast (*n* = 254)*	*P*†	*q*-Value‡
Age at time of admission (years)	71 (57, 80)	70 (59, 81)	>0.9	>0.9
Unknown	0	1		
Sex			>0.9	>0.9
Female	86 (55.84%)	142 (55.91%)		
Male	68 (44.16%)	112 (44.09%)		
BMI	26 (21, 29)	25 (21, 30)	>0.9	>0.9
Unknown	37	44		
CCI	4.00 (2.00, 6.00)	3.00 (2.00, 5.00)	0.045§	0.3
CFS			0.5	>0.9
CFS 1–3	84 (61.76%)	147 (63.09%)		
CFS 4–6	39 (28.68%)	71 (30.47%)		
CFS 7–9	13 (9.56%)	15 (6.44%)		
Unknown	18	21		
Previous episode of adhesive SBO	57 (37.01%)	93 (36.61%)	>0.9	>0.9
White blood cell count (×10^9^/l)	11.1 (8.6, 13.8)	10.7 (8.3, 13.9)	0.6	>0.9
Unknown	0	1		
C-reactive protein (mg/l)	10 (5, 35)	13 (4, 46)	0.3	>0.9
Unknown	2	3		
Albumin (g/dl)	41 (38, 45)	40 (36, 44)	0.063	0.3
Unknown	3	4		
Lactate (mmol/l)	1.60 (1.10, 2.40)	1.40 (1.10, 2.03)	0.4	>0.9
Unknown	55	82		
AKI at admission	28 (18.18%)	47 (18.58%)	>0.9	>0.9
Unknown	0	1		

AKI, acute kidney injury; CCI, Charlson Co-morbidity Index; CFS, Clinical Frailty Score; SBO, small bowel obstruction. *Median (i.q.r.); *n* (%). ^†^Wilcoxon rank sum test; Pearson’s Chi-squared test. ^‡^False discovery rate correction for multiple testing. ^§^Statistically significant.

### Contrast challenge

Of the 408 patients who underwent an initial trial of NOM, 254 (62.3%) received a WSCA challenge and 154 (37.7%) did not. Gastrografin was the most common WSCA used (*n* = 229, 90.5%), at 100 ml dose (96.9%) (Supplementary Material  *S2*). Other agents used were Omnipaque (*n* = 7, 2.8%) and Gastromiro (*n* = 17, 6.7%).

Patients receiving WSCA had lower CCIs compared to those who did not (3.0 *versus* 4.0, *P* = 0.041); however, this did not remain statistically significant after multiple testing correction (*q* = 0.3). There were no other differences in patient characteristics observed. The median time from admission to WSCA administration was 1.0 day (i.q.r. 0.0–1.3).

**Fig. 2:**
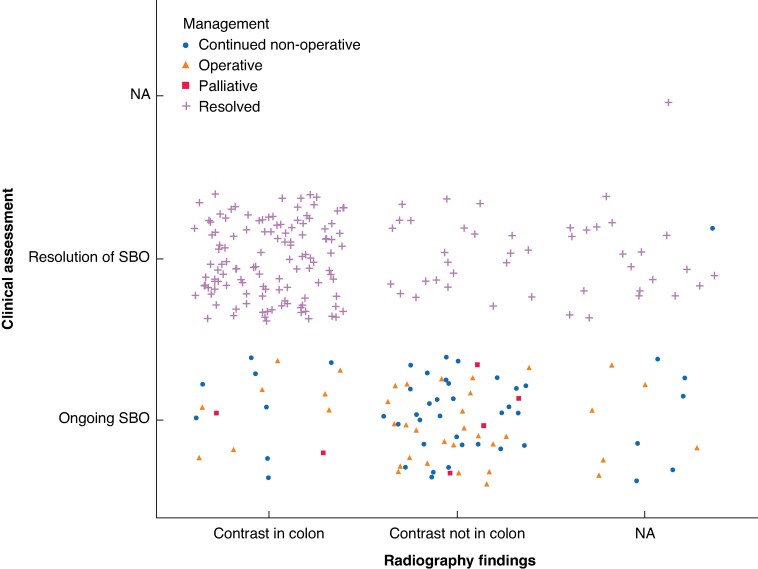
Plot comparing management strategies following initial contrast challenge and their associated clinical and radiographic findings NA, not applicable; SBO, small bowel obstruction.

Of those who had a WSCA challenge, 58.1% had a follow-up AXR within 8 h (*n* = 147) (Supplementary Material  *S3*). Following the first WSCA challenge, 163 cases (64.2%) of aSBO resolved, with contrast observed in the colon in 119 cases (83.8%). SBO persisted in 91 cases (35.8%), of which 39 (42.9%) progressed to surgery and 46 (50.5%) continued with NOM. There were radiographic findings of contrast in the colon in 24.2% (*n* = 8) of those progressing to operative management and in 20.5% (*n* = 8) of those with continued NOM (*Fig. 2*, Supplementary Material  *S4*).

A second WSCA challenge was performed in 26 patients (26.8%), with resolution in 8 (30.8%) and ongoing aSBO in 18 (69.2%). Twelve patients (66.7%) progressed to surgery at this point and six (33.3%) continued with NOM. A third contrast challenge was performed in three patients (16.7%), with resolution in one and ongoing aSBO in the remaining two. All repeat WSCA challenges were performed with Gastrografin and showed significantly lower rates of resolution (*P* < 0.001).

### Success of non-operative management and operative intervention

Of the 408 patients initially managed with NOM for aSBO, 81.6% (*n* = 333/408) successfully resolved with NOM, and 18.4% (*n* = 75/408) ultimately required surgery. The success rate of NOM was higher in those managed without a WSCA challenge compared to those who received one (83.8% *versus* 73.2%, *P* = 0.014). Additionally, the operative rates were higher in NOM patients who received a WSCA challenge compared to those who did not (22.4% *versus* 11.7%, *P* = 0.007; *Table 2*). Of the 220 patients undergoing active NOM with contrast challenge, contrast in the colon predicted resolution with a sensitivity of 66.5% (95% c.i. 59.0 to 73.3%), and a specificity of 56.1% (95% c.i. 39.7% to 71.5%).

**Table 2:** Comparison of clinical outcomes of non-operative management in patients with and without a contrast challenge

Characteristic	No contrast (*n* = 154)*	Contrast (*n* = 254)*	*P*†	*q*-Value‡
Operation for SBO	18 (11.69%)	57 (22.44%)	**0.007**§	**0**.**037**§
Time to GI recovery (days)	1 (1, 2)	2 (1, 5)	**<0**.**001**§	**<0**.**001**§
Length of stay (days)	4 (2, 7)	6 (3, 12)	**<0**.**001**§	**<0**.**001**§
In-hospital death	9 (5.84%)	13 (5.12%)	0.8	>0.9
Urinary tract infection	4 (2.60%)	8 (3.15%)	>0.9	>0.9
Pneumonia	15 (9.74%)	34 (13.39%)	0.3	0.6
Cardiac	4 (2.60%)	13 (5.12%)	0.2	0.5
DVT/PE	2 (1.30%)	4 (1.57%)	>0.9	>0.9
Delirium	7 (4.55%)	14 (5.51%)	0.7	>0.9
Intra-abdominal sepsis	4 (2.60%)	6 (2.36%)	>0.9	>0.9
Radiological drainage	0 (0.00%)	2 (0.79%)	0.5	>0.9
Unplanned HDU/ICU admission	3 (1.95%)	13 (5.12%)	0.11	0.3
Superficial surgical site infection	0 (0.00%)	3 (5.26%)	>0.9	>0.9
Abdominal wall dehiscence	0 (0.00%)	1 (1.75%)	>0.9	>0.9
Anastomotic leak				
No	18 (100.00%)	57 (100.00%)		
Reoperation	1 (5.56%)	2 (3.51%)	0.6	>0.9
30-day readmission	27 (17.76%)	25 (9.84%)	**0**.**021**§	0.088
Subsequent operation for index NOM admission	4 (2.94%)	0 (0.00%)	**0**.**027**§	0.092

DVT, deep vein thrombosis; HDU, high-dependency unit; ICU, intensive care unit; PE, pulmonary embolism; SBO, small bowel obstruction. *n (%). ^†^Pearson’s Chi-squared test; Fisher’s exact test. ^‡^False discovery rate correction for multiple testing. ^§^Statistically significant.

The most common operative approach was open (*n* = 44/75, 58.7%) and level of obstruction was at the ileum (*n* = 40/75, 53.3%). Patients who received a contrast challenge had a median longer time to operation (3.0 days, i.q.r. 2.0–5.0 *versus* 1.0 day, i.q.r. 1.0–1.0, *P* < 0.001). Overall reported rates of intraoperative bowel ischaemia were 19/75 (25.3%); however, there was no significant difference in rates of ischaemia or small bowel resection between the contrast and non-contrast groups (Supplementary Material  *S5*).

**Fig. 3:**
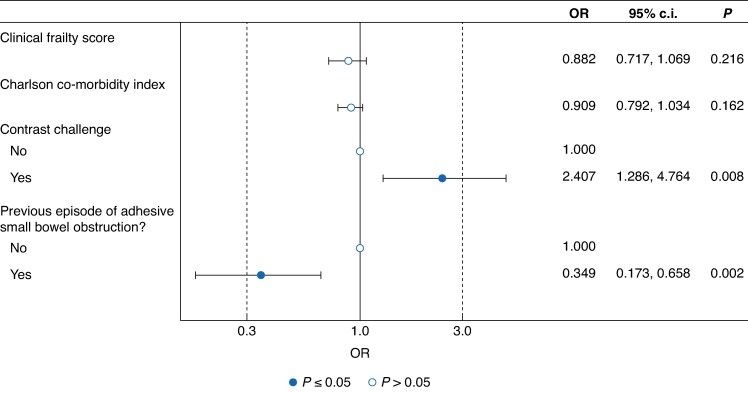
Regression model exploring the impact of frailty, co-morbidities, prior small bowel obstruction, and use of contrast on rates of surgery

A regression model was developed to explore the impact of frailty, co-morbidities, prior SBO, and use of contrast on rates of surgery (*Fig. 3*). The final model AIC was 327.7. It demonstrated that increased frailty and CCI had a non-significant trend to reduced rates of surgery. Previous episodes of SBO were associated with lower rates of surgery (OR 0.35 (95% c.i. 0.17 to 0.66), *P* = 0.002), and use of contrast was associated with increased rates of surgery (OR 2.41 (95% c.i. 1.29 to 4.76), *P* = 0.008).

### Clinical outcomes

A summary of outcomes in NOM patients is displayed in *Table 2*, with outcomes stratified by type of contrast agent in Supplementary Material  *S6*.

The overall in-hospital mortality rate in this cohort was 5.4% (*n* = 22/408). Following an initial WSCA challenge, six of the patients were subsequently palliated and two of the patients who died received a further second WSCA challenge. Of the patients who died, 18.2% (*n* = 4/22) had an operation. There were no significant differences in in-hospital death between those who received and did not receive a WSCA challenge, both for all NOM patients (5.1% *versus* 5.8%, *P* = 0.8) and those who went on to receive an operation (3.5% *versus* 11.1%, *P* = 0.2).

The median time to GI recovery was shorter in the no-contrast group at 1 day (i.q.r. 1–2) *versus* 2 days (i.q.r. 1–5) for the contrast group (*P* < 0.001). The median hospital length of stay was 4 days (i.q.r. 2–7) for the non-contrast group *versus* 6 days (i.q.r. 3–12) for the contrast group (*P* < 0.001). Similarly, among patients who underwent surgery, those who received a contrast challenge had a longer median hospital length of stay (13 days, i.q.r. 9–24 *versus* 7 days, i.q.r. 6–15, *P* = 0.033, *q* = 0.4; Supplementary Material  *S7*).

The most common complications reported in this cohort were pneumonia (*n* = 49/408, 12.0%), delirium (*n* = 21/408, 5.1%), cardiac (*n* = 17/408, 4.2%), and unplanned high-dependency unit/intensive care unit admission (*n* = 16/408, 3.9%). There were no significant differences in complication rates between those who did and did not have a WSCA challenge (Supplementary Material  *S7*).

Fifty-two patients (12.7%) were readmitted within 30 days following discharge after an initial trial of NOM for aSBO; five (9.6%) underwent surgery during their index admission, whereas 47 (90.4%) did not (Supplementary Material  *S8*). Patients who did not receive WSCA had a higher 30-day readmission rate (17.8% *versus* 9.8%, *P* = 0.021), and were more likely to have a subsequent operation (2.9% *versus* 0.0%, *P* = 0.027; *Table 2*). However, after multiple testing correction, these associations did not reach statistical significance (*q* = 0.088 and *q* = 0.092 respectively). The most common reason for readmission was recurrent SBO (*n* = 31/52, 59.6%), which was significantly more frequent in patients managed non-operatively without WSCA (76.0% *versus* 45.5%). Non-SBO-related reasons, such as infections and deconditioning, were more common among those managed non-operatively with WSCA (54.5% *versus* 24.0%, *P* = 0.032).

## Discussion

This study provides prospective multicentre ‘real-life’ data on the NOM of patients presenting with aSBO. The overall success of NOM was 81.6%. The use of WSCA was widespread despite a reported shortage, with 62.3% of patients receiving a contrast challenge. However, repeated contrast challenges showed diminishing resolution rates, suggesting limited therapeutic benefit beyond initial administration.

A comparison of this cohort to previous UK reports provides an interesting contrast. The NASBO (National Audit of Small Bowel Obstruction) report showed that 31.0% of patients with aSBO received WSCA^15^ compared to 62.3% in the present study, reflecting a broader adoption of WSCA in aSBO management. This finding underscores the pragmatic and adaptable nature of clinical practice, where hospitals and clinicians likely implemented local strategies to maintain access to contrast agents despite reported supply chain challenges. Although it is difficult to determine the precise impact of the perceived shortage on decision-making, this study highlights how real-world data collection can provide unexpected insights into resource utilization and the resilience of clinical workflows during periods of uncertainty. However, one finding that has remained constant is the high rate of plain abdominal radiography prior to CT, contrary to recommendations from the National Confidential Enquiry into Patient Outcome and Death and the American College of Radiology, which advocate for early CT as the preferred diagnostic modality^16,17^.

This increased use of WSCA *versus* NASBO^15^ is accompanied by notable variations in clinical practice. This includes differences in the use of AXR post-contrast, as highlighted here. Other variations, such as the management of nasogastric drainage and the spigotting of nasogastric tubes, have been highlighted in previous trials^18–20^. The impact of these variations on the therapeutic or stratifying efficacy of WSCA remains unclear and may reflect concerns about perceived risks, such as aspiration pneumonia.

Additionally, there is inconsistency in the timing and utility of follow-up AXRs. If contrast fails to reach the colon within 24 h, this is highly indicative of NOM failure^21^. However, the optimal timing for follow-up imaging remains debated. Delaying AXR to 12 h post-WSCA administration may offer limited additional sensitivity and specificity while potentially delaying necessary surgical intervention^22^. An earlier AXR (6–8 h) might be advisable to balance this, by minimizing delays without compromising diagnostic value. Among patients who had resolved aSBO following a WSCA challenge, 83.8% demonstrated radiographic evidence of contrast in the colon. However, radiographic evidence of contrast in the colon was also observed in one-fifth of those with ongoing aSBO, highlighting a mismatch between radiographic and clinical findings. This raises concern about the reliability of AXR as a tool to guide decision-making. The sensitivity and specificity of contrast in the colon in the prediction of resolution of aSBO is much lower than that reported in the 2007 Cochrane review^22^. Given these inconsistencies and potential delays, there is a strong case for moving away from routine follow-up AXRs in favour of clinical decision-making guided by patient symptoms and clinical progression.

The physiological rationale for WSCA use in aSBO extends beyond its established diagnostic role. However, although these effects are frequently cited in clinical practice, there is a paucity of direct experimental evidence supporting a consistent therapeutic benefit. The first round of contrast challenges was associated with a two-thirds rate of resolution. For subsequent challenges, this drops to one-third. This suggests that any potential osmotic effects may be limited to initial administration rather than cumulative impact. Repeat challenges may delay further decision-making and increase the risk of adverse events. Interestingly, among patients who did not achieve resolution after repeated challenges, around half proceeded to surgery, whereas the other half continued with NOM. This pattern suggests that WSCA may often be used to defer or avoid surgery, rather than to stratify patients effectively based on the initial challenge outcome. The impact of repeated challenges remains unexplored in trials, but the relatively consistent resolution rate of approximately 30% after subsequent attempts suggests limited benefit. Therefore, a ‘one and done’ approach might be advocated, wherein a single WSCA challenge is performed, followed by timely progression to definitive management if resolution is not achieved.

Modelling revealed a positive association between the use of WSCA and the likelihood of undergoing surgery. This may mean that early contrast is deployed to facilitate early identification of those at high risk of NOM failure. Another suggestion is that it may be employed as a strategy to defer or potentially avoid an operation, as evidenced by its use in patients who were ultimately palliated following a failed challenge. Notably, patients who had previous episodes of aSBO demonstrated lower rates of surgery. This observation may reflect the effective resolution of obstruction through non-operative means in selected cases. Although the regression model adjusted for key baseline clinical factors, the potential for selection bias remains.

Alternative contrast agents are widely used diagnostically. Low-osmolar WSCA such as Omnipaque are often recommended for patients at higher aspiration risk due to their reduced potential to induce an inflammatory response in the lungs^16,23^. Previous cohorts investigating the use of Omnipaque as aSBO treatment indicate its safety and efficacy^24,25^, similar to observed in the present study. However, the limited number of patients receiving alternative contrast agents in this study limits definitive conclusions, and further research is needed to directly compare the clinical outcomes of different contrast agents in aSBO.

A key risk of NOM is the potential for bowel ischaemia. In the present cohort, 25.3% of patients who underwent surgery after an initial trial of NOM had intraoperative evidence of ischaemia, reinforcing the importance of careful patient selection and close monitoring. Emergent surgery is necessary for patients with clinical or radiological signs suggestive of bowel ischemia^26^. Although delaying surgery may allow resolution in select cases, prolonged NOM in the presence of ongoing obstruction increases the risk of irreversible bowel injury. Notably, the rate of ischaemia did not significantly differ between those who received WSCA and those who did not, suggesting that contrast administration itself does not exacerbate this risk. However, the longer time to surgery observed in the WSCA group highlights the need for a clear threshold for when NOM should be considered unsuccessful.

The study is not without limitations. Its observational design means the findings are of association only, and not causation. Patients selected for WSCA challenges did not have lower CCI, and experienced higher rates of surgery and longer times to operation. This means surgeons are potentially deferring surgery in cases where it is ultimately anticipated, introducing selection bias. Time to GI-2 was recorded in days rather than hours. Although this provides a practical estimate, this may limit precision in assessing subtle differences in recovery time. The 30-day follow-up period is relatively short, and the absence of patient-reported outcomes measures (PROMs) limits insight into the impact of treatment strategies from the patient’s perspective. Additionally, data on operative details and ischaemia were poorly reported, as was the documentation of CFS data. The impact of aSBO and its management on quality of life, symptom burden, and post-discharge recovery remains underexplored in the current literature. The PRO-diGI PROM for gastrointestinal recovery has been developed for use in this population^27^, and might be utilized in future studies.

However, the study’s strengths lie in its modern, prospective, and multicentre nature, which is necessary for capturing variations in practice. This approach has generated testable hypotheses and provided valuable insights into how surgeons have translated data from clinical trials into real-world practice. Although sites were provided with general guidance, contrast administration and follow-up protocols were not strictly standardized, which may have introduced variability in practice. Furthermore, the data collected were unable to fully capture factors such as the presence of a hostile abdomen, which may have influenced treatment decisions. A recent survey of surgeons examining their beliefs and rationale for WSCA use found considerable divergence in opinions about its primary mode of action—whether diagnostic, therapeutic, or both^6^. These findings highlight the need for greater standardization in contrast use protocols and a clearer understanding of when and why WSCA is employed in clinical practice. Future research should explore patient-reported outcomes and alternative contrast agents to further optimize the management of aSBO.

## Collaborators

Josephine Walshaw, Daniel Ashmore, Adam Peckham-Cooper, Matthew J. Lee, Raimundas Lunevicius, Adam Daniel Gerrard, Jay Roe Tan, Ross Nieuwoudt, Jacob Mewse, Alice Luesley, Nicola Eardley, Rachael Clifford, Lucia Sepesiova, Tim Wilson, Thomas Hall, Shoieb Mridha, Jessie Blackburn, Ajay Belgaumkar, Khaldoun Fozo, Sukanya Thavanesan, Oroog Ali, Alex McCulla, Ross Lilley, Corin Lathan, Rory Austin, Chathura Munasinghe, Milad Tavakoli, Ademola Adeyeye, Amyn Haji, Ayinke Dosu, Kasthoory Kandiah, Panagiotis Kapsampelis, Ioannis Gerogiannis, Georgios Bointas, Negar Ghaffari, Shirley Chan, Keshav Jindal, Danny Lamdin, Devika Nair, Sita Kotecha, Ala Saab, Alexander Wilkins, Annabelle Williams, Naomi Warner, Anamaria Schipor, Liam Martin, Ashuvini Mehendran, Marianne Hollyman, Ahmed Abdal Rahim, Mike Richardt, John Wayman, Hannah Dunlop, Ning Xuan Ho, Olivia Cory, Michael Okocha, Alasdair Ralston, Luke Williams, Michael El-Boghdady, Hussayn Shinwari, Edward J Nevins, Kohei Yamada, Aya Musbahi, Tejinderjit Athwal, Alex Carney, Daisy Evans, Tomasz Galus, Victoria Gregory, Sam Jacobs, Nicholas Newton, Andrew Daley.

## Supplementary Material

znaf104_Supplementary_Data

## Data Availability

The data underlying this article will be shared on reasonable request to the corresponding author.
