# Supplementary material for: SBO ACTION: conservative Small Bowel Obstruction management in the Absence of standard ConTrast agents ON outcomes
Source: Br J Surg. 2025 Jun 24;112(6):znaf104. doi: 10.1093/bjs/znaf104 (PMC12206153; doi:10.1093/bjs/znaf104)
Supplement: znaf104_Supplementary_Data [file znaf104_supplementary_data.docx]

**SBO ACTION: conservative Small Bowel Obstruction management in the Absence of standard ConTrast agents ON outcomes**

**Authors:** SBO ACTION Collaborative*

*A full list of collaborating authors is shown under the heading ‘*SBO ACTION Collaborative*’

**Corresponding Author:**

Josephine Walshaw

j.b.walshaw@leeds.ac.uk

Leeds Institute of Medical Research

St James’s University Hospital

University of Leeds

Beckett Street

Leeds

LS9 7TF

ORCID: 0000-0003-0450-273X

Twitter: @josiewalshaw1

**Supplementary Materials - Index**

| **Supplementary Appendixes** |  |
| --- | --- |
| SBO ACTION Collaborative | *pag. 2* |
| Suppl 1 - Case Report Form | *pag. 3* |
| **Supplementary Figures and Tables** |  |
| Suppl 2 - Volume of contrast agents used during the first contrast agent challenge | *pag. 12* |
| Suppl 3 - Timing of abdominal x-ray following contrast challenge | *pag. 13* |
| Suppl 4 - Management strategies and their associated clinical and radiographic findings | *pag. 14* |
| Suppl 5 - Comparison of operative data in patients initially managed non-operatively with and without a contrast challenge | *pag. 15* |
| Suppl 6 - Outcomes of non-operative management, stratified by contrast type | *pag. 16* |
| Suppl 7 - Comparison of operative outcomes in patients initially managed non-operatively with and without a contrast challenge | *pag. 17* |
| Suppl 8 - Readmission data, stratified by contrast challenge administration and operative intervention following initial non-operative management | *pag. 18* |

**Supplementary Appendixes**

**SBO ACTION Collaborative**

***Steering Group (*denotes first author, ^†^denotes senior author):***

Josephine Walshaw*, Daniel Ashmore, Adam Peckham-Cooper, Matthew J Lee**^†^**

***Collaborating Authors (*denotes local principal investigator):***

Raimundas Lunevicius*, Adam Daniel Gerrard, Jay Roe Tan, Ross Nieuwoudt (**Aintree University Hospital, Liverpool, UK**); Jacob Mewse*, Alice Luesley (**Barnsley Hospital NHS Foundation Trust, Barnsley, UK**); Nicola Eardley*, Rachael Clifford, Lucia Sepesiova (**Countess of Chester Hospital NHS Foundation Trust, Chester, UK**); Daniel Ashmore*, Tim Wilson, Thomas Hall, Shoieb Mridha (**Doncaster and Bassetlaw Teaching Hospitals NHS Foundation Trust, Doncaster, UK**); Jessie Blackburn*, Ajay Belgaumkar, Khaldoun Fozo, Sukanya Thavanesan (**East Surrey Hospital, Surrey, UK**); Oroog Ali*, Alex McCulla, Ross Lilley (**Gateshead Health NHS Foundation Trust, Gateshead, UK**); Corin Lathan*, Rory Austin, Chathura Munasinghe, Milad Tavakoli, Josephine Walshaw, Adam Peckham-Cooper (**Leeds Teaching Hospitals NHS Trust, Leeds, UK**); Ademola Adeyeye*, Amyn Haji, Ayinke Dosu, Kasthoory Kandiah (**King’s College Hospital, London, UK**); Panagiotis Kapsampelis*, Ioannis Gerogiannis, Georgios Bointas, Negar Ghaffari (**Kingston Hospital NHS Foundation Trust, Kingston upon Thames, UK**); Shirley Chan*, Keshav Jindal, Danny Lamdin, Devika Nair (**Medway NHS Foundation Trust, Kent, UK**); Sita Kotecha*, Ala Saab, Alexander Wilkins (**Mid Yorkshire Teaching NHS Trust, Wakefield, UK**); Annabelle Williams*, Naomi Warner, Anamaria Schipor, Liam Martin (**Milton Keynes University Hospital, Milton Keynes, UK**); Ashuvini Mehendran*, Marianne Hollyman, Ahmed Abdal Rahim, Mike Richardt (**Musgrove Park Hospital, Taunton, UK**); John Wayman*, Hannah Dunlop, Ning Xuan Ho, Olivia Cory (**North Cumbria Integrated Care NHS Trust, Carlisle, UK**); Michael Okocha*, Alasdair Ralston, Luke Williams (**Royal United Hospitals Bath NHS Foundation Trust, Bath, UK**); Michael El-Boghdady*, Hussayn Shinwari (**St George’s University Hospitals NHS Foundation Trust, London, UK**); Edward J Nevins*, Kohei Yamada, Aya Musbahi (**Sunderland Royal Hospital, Sunderland, UK**); Tejinderjit Athwal*, Alex Carney, Daisy Evans, Tomasz Galus (**University Hospitals of North Midlands NHS Trust, Stoke-on-Trent, UK**); Matthew J Lee*, Victoria Gregory, Sam Jacobs, Nicholas Newton, Andrew Daley (**Queen Elizabeth Hospital Birmingham, Birmingham, UK**)

**Supplementary Material 1: Care Report Form**

| **Admission data** | | | |
| --- | --- | --- | --- |
| Q1 | Record ID (Generated by REDCap) |  | |
| Q2 | Age at time of admission (years) |  | |
| Q3 | Sex | Male Female | |
| Q4 | Height (cm) |  | |
| Q5 | Weight (Kg) |  | |
| Q6 | Comorbidities (select all that apply) | IHD  CCF  CVA  PVD  Mild liver disease  Severe liver disease  Peptic ulcer disease  DM (uncomplicated)  DM (complicated) | CKD  Dementia  Solid tumour  Metastatic tumour  Leukaemia  Lymphoma  Connective tissue disorder  Hemiplegia  AIDS |
| Q7 | What is the patient’s Rockwood clinical frailty score? | 1 / 2 / 3 / 4 / 5 / 6 / 7 / 8 / 9 | |
| Q8 | Previous episode of adhesive small bowel obstruction? | Yes No | |
|  | If yes, number of previous admissions with adhesive small bowel obstruction? | 1 / 2 / 3 / 4 / 5+ / unknown | |
| Q9 | Source of referral (Select one) | Emergency Department  General Practice  Referral from inpatient team  Other | |
|  | If other, please state |  | |
| Q10 | Date admitted to hospital | DD/MM/YY | |
| Q11 | Date first seen by a member of the surgical team | DD/MM/YY | |
| Q12 | Aetiology | Congenital band adhesion  Post-operative adhesions | |
| Q13 | Initial management strategy | Non-operative  Operative (decision made within 24 hours of admission)  Palliative | |

| **Baseline physiology** | | |
| --- | --- | --- |
| Please respond to the following questions using the lab results from the point closest to admission | | |
| Q14 | White Cell Count (x10^9^/L) |  |
| Q15 | C-Reactive Protein (mg/L) |  |
| Q16 | Albumin (g/dL) |  |
| Q17 | Lactate (mmol/L) |  |
| Q18 | Did the patient have an AKI at admission? | Yes No |

| **Diagnostic tests** | | |
| --- | --- | --- |
| Q19 | Abdominal X-ray performed prior to CT scan/ as initial imaging investigation | Yes No |
|  | If yes, date of abdominal X-ray | DD/MM/YY |
| Q20 | Abdominal CT scan performed | Yes No |
|  | If yes, date of abdominal CT | DD/MM/YY |

| **Contrast** | | |
| --- | --- | --- |
| Q21 | Did the patient receive a water-soluble contrast agent (e.g. gastrografin) and/or osmotic agent (e.g. mannitol) challenge as part of non-operative management, apart from when undergoing the initial CT scan? | Contrast / Osmotic / Both / No |
| Q22 | What date did the patient receive a water-soluble contrast and/or osmotic agent challenge? | DD/MM/YY |
| Q23 | What water-soluble contrast and/or osmotic agent was used? (tick all that apply) | Gastrografin  Omnipaque  Visipaque  Urografin  Mannitol  Other |
|  | If other, please state |  |
| Q24 | What water-soluble contrast and/or osmotic agent dose was used? (ml) |  |
| Q25 | How long after ingestion of the water-soluble contrast and/or osmotic agent was a follow-up radiograph performed? | <8 hours  8-16 hours  16-24 hours  >24 hours  Not performed |
|  | If performed, what were the follow-up radiograph results? | Contrast in colon  Contrast not in colon |
| Q26 | What was the clinical outcome of receiving the water-soluble contrast and/or osmotic agent challenge? | Resolution of SBO  Ongoing SBO |
| Q27 | If ongoing SBO, what was the next management strategy following initial non-operative management? | Continued non-operative  Operative  Palliative |
|  | | |
| Q28 | Was a second water-soluble contrast agent (e.g. gastrografin) and/or osmotic agent (e.g. mannitol) challenge performed as part of continued non-operative management, apart from when undergoing the initial CT scan? | Contrast / Osmotic / Both / No |
| Q29 | What date did the patient receive a water-soluble contrast and/or osmotic agent challenge? | DD/MM/YY |
| Q30 | What water-soluble contrast and/or osmotic agent was used? (tick all that apply) | Gastrografin  Omnipaque  Visipaque  Urografin  Mannitol  Other |
|  | If other, please state |  |
| Q31 | What water-soluble contrast and/or osmotic agent dose was used? (ml) |  |
| Q32 | How long after ingestion of the water-soluble contrast and/or osmotic agent was a follow-up radiograph performed? | <8 hours  8-16 hours  16-24 hours  >24 hours  Not performed |
|  | If performed, what were the follow-up radiograph results? | Contrast in colon  Contrast not in colon |
| Q33 | What was the clinical outcome of receiving the water-soluble contrast and/or osmotic agent challenge? | Resolution of SBO  Ongoing SBO |
| Q34 | If ongoing SBO, what was the next management strategy following initial non-operative management? | Continued non-operative  Operative  Palliative |
|  | | |
| Q35 | Was a third water-soluble contrast agent (e.g. gastrografin) and/or osmotic agent (e.g. mannitol) challenge performed as part of continued non-operative management, apart from when undergoing the initial CT scan? | Contrast / Osmotic / Both / No |
| Q36 | What date did the patient receive a water-soluble contrast and/or osmotic agent challenge? | DD/MM/YY |
| Q37 | What water-soluble contrast and/or osmotic agent was used? (tick all that apply) | Gastrografin  Omnipaque  Visipaque  Urografin  Mannitol  Other |
|  | If other, please state |  |
| Q38 | What water-soluble contrast and/or osmotic agent dose was used? (ml) |  |
| Q39 | How long after ingestion of the water-soluble contrast and/or osmotic agent was a follow-up radiograph performed? | <8 hours  8-16 hours  16-24 hours  >24 hours  Not performed |
|  | If performed, what were the follow-up radiograph results? | Contrast in colon  Contrast not in colon |
| Q40 | What was the clinical outcome of receiving the water-soluble contrast and/or osmotic agent challenge? | Resolution of SBO  Ongoing SBO |
| Q41 | If ongoing SBO, what was the next management strategy following initial non-operative management? | Continued non-operative  Operative  Palliative |

| **Operative management** | | |
| --- | --- | --- |
| Q42 | Did the patient undergo an operation for small bowel obstruction? | Yes No |
| Q43 | What date was the operation performed? | DD/MM/YY |
| Q44 | What was the NELA risk score? |  |
| Q45 | What operative approach was used? | Laparoscopic  Laparoscopic converted to open  Open |
| Q46 | What was the level of obstruction | Jejunum / ileum / not stated |
| Q47 | Small bowel ischaemia description | Reversible / irreversible / no ischaemia / not stated |
| Q48 | Procedure details  (select all that apply) | Division (single) band adhesion  Adhesiolysis  Small bowel resection  Formation jejunostomy  Formation ileostomy  Anastomosis of bowel  Other |
|  | If other, please state |  |

| **Care episode data** | | | | | |
| --- | --- | --- | --- | --- | --- |
| Q49 | Date patient first tolerated solid food | DD/MM/YY | | | |
| Q50 | Date patient first passed flatus or defecated | DD/MM/YY | | | |
| Q51 | In-hospital death | Yes No | | | |
| Q52 | Date of discharged or in-hospital death | DD/MM/YY | | | |
| Q53 | Was the patient readmitted within 30-days following discharge? | Yes No |  |  |  |
| Q54 | Reason for readmission | Further episode of SBO  Complication of surgery  Other | | | |
|  | If other, please state |  | | | |
| Q55 | In patients that didn’t have an operation in their initial admission, was an operation performed for small bowel obstruction within 30-days following discharge? | Yes No | | | |

| **Did the following complications of management occur whilst hospital inpatient?** | | | |
| --- | --- | --- | --- |
| Q56 | UTI | Yes No |  |
| Q57 | Pneumonia | Yes No |  |
| Q58 | Cardiac | Yes No |  |
| Q59 | DVT/PE | Yes No |  |
| Q60 | Delirium | Yes No |  |
| Q61 | Intra-abdominal sepsis | Yes No |  |
| Q62 | Radiological drainage | Yes No |  |
| Q63 | Unplanned HDU/ITU admission | Yes No |  |
| Q64 | If operative management, Superficial Surgical Site infection (SSI) | Yes No |  |
| Q65 | If operative management, Abdominal Wall dehiscence | Yes No |  |
| Q66 | If operative management, Anastomotic leak | Yes No |  |
| Q67 | If operative management, Reoperation | Yes No | |
|  | Primary indication for reoperation |  | |

**Care Report Form Definitions**

**Admission data**

Q1. This number is generated by REDCap when you begin entering data. Please keep a record locally which can be cross-referenced with your local data.

Q2. Age in completed years on the date of admission to hospital.

Q3. Please indicate sex of the patient.

Q4. Height on admission in centimetres, rounded to the nearest centimetre. If height only available in feet and inches, please convert using an online calculator.

Q5. Weight on admission in kilograms, rounded to the nearest kilogram. If only available in stones and pounds, please convert using an online calculator.

Q6. These are comorbidities as defined by the Charlson Comorbidity Index. Each should be marked as present if there is any previous documented history of each diagnosis.

| Myocardial infarct (MI) | History of medically documented myocardial infarction |
| --- | --- |
| Congestive heart failure (CCF) | Symptomatic congestive heart failure w/ response to specific treatment |
| Peripheral vascular disease (PVD) | Intermittent claudication, peripheral. Arterial bypass for insufficiency, gangrene, acute arterial insufficiency, untreated aneurysm (>=6cm) |
| Cerebrovascular disease (CVA) (except hemiplegia) | History of TIA, or CVA with no or minor sequelae |
| Dementia | Chronic cognitive deficit |
| Chronic pulmonary disease (COPD) | Symptomatic dyspnoea due to chronic respiratory conditions (including asthma) |
| Connective tissue disease | SLE, polymyositis, polymyalgia rheumatica, moderate to severe rheumatoid arthritis |
| Peptic ulcer disease | Patients who have required treatment for peptic ulcer disease |
| Mild liver disease | Cirrhosis without portal hypertension, chronic hepatitis |
| DM (uncomplicated) | Diabetes with medication (including insulin) |
| DM (complicated) | Retinopathy, neuropathy, nephropathy |
| Hemiplegia (or paraplegia) | Hemiplegia or paraplegia |
| Moderate or severe renal disease | Creatinine >265 umol/l, dialysis, transplantation, uremic syndrome |
| Solid tumour (non-metastatic) | Initially treated in the last 5 years exclude non-melanomatous skin cancers and in situ cervical carcinoma |
| Leukaemia | CML, CLL, AML, ALL, PV |
| Lymphoma, Multiple Myeloma... | Non-Hodgkin’s Lymphoma, Hodgkin's, Waldenström, multiple myeloma |
| Moderate or severe liver disease | Cirrhosis with portal hypertension +/- variceal bleeding |
| Metastatic solid tumour | self-explanatory |
| AIDS | AIDS and AIDS-related complex Suggested: as defined in latest definition |

Q7. Please record the patient’s Rockwood clinical frailty score.

Q8a. Please state if the patient has had a previous radiological or intra-operative diagnosis of adhesive small bowel obstruction.

Q8b. Please state the number of previous admissions with adhesive small bowel obstruction.

Q9. Please indicate only one source of referral.

Via **Emergency Department** only applies if the patient was not referred to attend the hospital by the GP.

**General Practice** means direct acute referral to the hospital by the GP.

**Surgical clinic admission** means the patient was review by a doctor working in the trust and directly

referred to be admitted to hospital.

**Referral from inpatient team** means the patient was already an inpatient within the trust and after

admission has been referred to the general surgical team.

Q10. Date patient admitted to hospital, regardless of whether or not this was directly under the surgical

team.

Q11. Date first reviewed by any member of the surgical team. Only complete if different from Q8b.

Q12. Aetiology of small bowel obstruction – the cause of the obstruction as defined by clinical information or radiological imaging in non-operative cases, or intraoperative findings in those patients who are taken to surgery.

Q13. Was the initial management strategy within the first 24hrs of diagnosis non-operative/conservative (watch and wait, gastrografin etc.) or to list for operation (even if the operation was not performed in the first 24hrs), or palliative (symptomatic management only with no surgery intended at any point)?

**Baseline physiology**

Q14. Please give White Cell Count (x109/L) as measured at the closest point to or after admission. Please only use levels taken within the first 48 hours of admission and taken preoperatively.

Q15. Please give C-reactive protein (mg/L) as measured at the closest point to or after admission. Please only use levels taken within the first 48 hours of admission and taken preoperatively.

Q16. Albumin at admission (g/dL) – please use the first albumin level taken on admission to hospital. Please only use levels taken within the first 48 hours of admission and taken preoperatively. Please leave blank if no value available.

Q17. Please give Lactate (mmol/L) as measured at the closest point to or after admission. Please only use levels taken within the first 48 hours of admission and taken preoperatively. Please leave blank if no value available.

Q18. Please tick yes if there is evidence of one of; laboratory generated warning of kidney injury, blood creatinine level has risen from the baseline value by 26 µmol/L or more within 48 hours, blood creatinine level has risen by 50% or more within the past 7 days, patient is passing less than 0.5ml urine per kg per hour for more than 6 hours in the first 48 hours of admission.

**Diagnostic tests**

Q19. Was an Abdominal X-ray performed prior to CT scan of the abdomen, as the initial imaging investigation? If so, please give the date this was performed.

Q20. Was a CT scan of their abdomen performed prior to commencement of management? If so, please give the date this was performed.

**Contrast**

Q21. Was a water-soluble contrast agent (e.g. gastrografin) and/or osmotic agent (e.g. mannitol) used as part of non-operative management, apart from when undergoing the initial CT scan? Please indicate whether one, both, or neither of these agents were used

Q22. If so, please give the date this was performed.

Q23. What contrast/osmotic agent was used for this individual challenge? Please select all that apply.

Q24. What dose was used (in ml)? If multiple agents used for one challenge please state doses for each.

Q25a. Please indicate how long after ingestion (in hours) was a follow-up radiograph performed to assess the results of the challenge.

Q25b. If performed, please indicate whether the contrast reached the colon on the follow-up radiograph.

Q26. Please state whether the water-soluble contrast or osmotic agent challenge resolved the SBO.

Q27. If the patient had ongoing small bowel obstruction after received a water-soluble contrast agent, did the next step in management involve continued non-operative/conservative management (watch and wait, gastrografin etc.), or to list for an operation, or palliative (symptomatic management only with no surgery intended)?

Q28-34 Answer if a second water-soluble contrast agent was used

Q35-41 Answer if a third water-soluble contrast agent was used

**Operative management**

Q42. Did the patient undergo an operation/procedure for SBO? This includes any operation, radiological or endoscopic procedure performed with the intention of resolving the symptoms, not purely for diagnosis.

Q43. Please indicate the date this was performed.

Q44. Please state the National Emergency Laparotomy Audit (NELA) risk score, as calculated from https://data.nela.org.uk/riskcalculator/.

Q45. If the patient had an operation, state whether the procedure started as a laparoscopic procedure, open from start to finish, or laparoscopic converted to open during the procedure (including laparoscopic assisted).

Q46. What was the level of the small bowel obstruction? Jejunum or ileum.

Q47. Was there evidence of small bowel ischaemia. If yes, was this reversible or irreversible.

Q48. Please indicate each of the procedures that were carried out as part of the intervention.

**Care episode data**

Q49. Please indicate the date the patient first resumed solid food following resolution of their SBO.

Q50. Please indicate the date the patient first passed flatus or defecated following resolution of their SBO.

Q51. In hospital death – Please indicate whether the patient died prior to discharge from the acute hospital.

Q52. Please indicate the date of discharge from the hospital OR if the patient died prior to discharge from hospital please state the date of death.

Q53. Please indicate if the patient was readmitted within 30 days of discharge.

Q54 If applicable, please record the reason for readmission.

Q55. Please indicate if an operation was performed for small bowel obstruction within 30 days of discharge (in patients that did not have an operation in their initial admission).

**In hospital complications**

Q56-67. In hospital complications

| Urinary Tract Infection (UTI) | Must have both criteria:  (1) Patient has at least one of the following signs or symptoms: fever (>38.0°C); suprapubic tenderness; costovertebral angle pain or tenderness; urinary urgency; urinary frequency; dysuria  (2) Patient has a urine culture with no more than two species of organisms identified, at least one of which is a bacterium of ≥105 CFU/ml. |
| --- | --- |
| Pneumonia | Must meet one of the criteria:  (1) Rales or dullness to percussion on physical examination of chest and any of the following: new onset of purulent sputum or change in character of sputum; organism isolated from blood culture; isolation of pathogen from specimen obtained by transtracheal aspirate, bronchial brushing or biopsy.  (2) Chest radiographic examination shows new or progressive infiltrate, consolidation, cavitation or pleural effusion and any of the following: new onset of purulent sputum or change in character of sputum; organism isolated from blood culture; isolation of pathogen from specimen obtained by transtracheal aspirate, bronchial brushing or biopsy; isolation of virus or detection of viral antigen in respiratory secretions; diagnostic single antibody titre (IgM) or four-fold increase in paired serum samples (IgG) for pathogen. |
| Cardiac | All cardiac complications newly diagnosed whilst inpatient (e.g. atrial fibrillation, myocardial infarction, etc). |
| Deep Vein Thrombosis / Pulmonary Embolism | Radiologically confirmed whilst inpatient. |
| Delirium | Acute confusional state with change from the patient’s normal cognitive baseline |
| Intraabdominal sepsis | Must meet one of the criteria:  (1) A clinician diagnosis of wound infection with dehiscence of mass closure or any layer below fat/scarpa’s fascia  (2) A clinical diagnosis of intra-abdominal collection (fever/abdominal pain) with operative/radiological evidence of a collection |
| Radiological Drainage | Any additional radiological drainage procedures, including image-guided aspiration of collection or placement of a drain |
| Unplanned HDU/ITU admission | Any unplanned episodes, even if unrelated to the primary presentation. |
| Superficial Surgical Site Infection (SSI) | Must meet one of the criteria:  (1) Purulent drainage from the incision  (2) At least two of: pain or tenderness; localised swelling; redness; heat; fever; AND incision opened deliberately to manage infection or the clinician diagnoses a SSI  (3) Wound organisms AND pus cells from aspirate/swab |
| Abdominal Wall Dehiscence | Full thickness dehiscence of laparotomy wound whilst inpatient |
| Anastomotic Leakage | A clinical diagnosis will require symptoms related to leakage (gas, pus or faecal discharge from the drainage site, peritonitis or discharge of pus from the rectum). In the event of a clinically suspicious leak (fever or abdominal pain) the diagnosis can be established by operative or radiological diagnosis. When an anastomosis is defunctioned the presence or absence of a leak will be established by contrast radiology. |
| Reoperation | Any return to theatre for a general surgical cause whilst inpatient. Please state the primary indication for reoperation. |

**Supplementary Figures and Tables**

**Supplementary Material 2: Volume of contrast agents used during the first contrast agent challenge. (ml = millilitres).**

| **Volume (ml)** | **Gastrografin**  N = 229 ^1^ | **Omnipaque**  N = 7 ^1^ | **Other**  N = 17 ^1^ |
| --- | --- | --- | --- |
| 10 | 0 (0.00%) | 2 (28.57%) | 0 (0.00%) |
| 50 | 2 (0.88%) | 2 (28.57%) | 17 (100.00%) |
| 100 | 219 (96.90%) | 2 (28.57%) | 0 (0.00%) |
| 150 | 4 (1.77%) | 0 (0.00%) | 0 (0.00%) |
| 200 | 1 (0.44%) | 0 (0.00%) | 0 (0.00%) |
| 400 | 0 (0.00%) | 1 (14.29%) | 0 (0.00%) |
| Unknown | 3 | 0 | 0 |
| ^1^n (%) | | | |
|  | | | |

**Supplementary Material 3: Timing of abdominal x-ray (AXR) following contrast challenge: Not performed (n=30), <8 hours (n=147), 8-16 hours (n=29), 16-24 hours (n=22), >24 hours (n=22).**

*
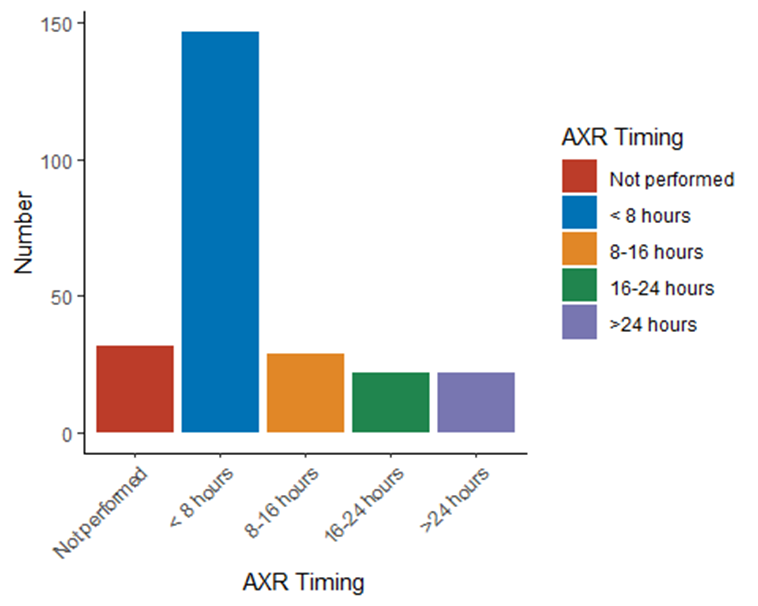
*

**Supplementary Material 4: Management strategies and their associated clinical and radiographic findings. (SBO = Small Bowel Obstruction).**

| **Characteristic** | **Continued non-operative**  N = 46^1^ | **Operative**  N = 39^1^ | **Palliative**  N = 6^1^ | **Resolved**  N = 163^1^ | **p-value**^2^ | **q-value^3^** |
| --- | --- | --- | --- | --- | --- | --- |
| Radiograph findings |  |  |  |  | **<0.001*** | **<0.001*** |
| Contrast in colon | 8 (20.51%) | 8 (24.24%) | 2 (33.33%) | 119 (83.80%) |  |  |
| Contrast not in colon | 31 (79.49%) | 25 (75.76%) | 4 (66.67%) | 23 (16.20%) |  |  |
| Unknown | 7 | 6 | 0 | 21 |  |  |
| Clinical assessment |  |  |  |  | **<0.001*** | **<0.001*** |
| Ongoing SBO | 45 (97.83%) | 39 (100.00%) | 6 (100.00%) | 0 (0.00%) |  |  |
| Resolution of SBO | 1 (2.17%) | 0 (0.00%) | 0 (0.00%) | 162 (100.00%) |  |  |
| Unknown | 0 | 0 | 0 | 1 |  |  |
| ^1^n (%) | | | | | | |
| ^2^Fisher's exact test | | | | | | |
| ^3^False discovery rate correction for multiple testing | | | | | | |
| *Statistically significant | | | | | | |

**Supplementary Material 5: Comparison of operative data in patients initially managed non-operatively with and without a contrast challenge. (CFS = Clinical Frailty Score, NELA = National Emergency Laparotomy Audit).**

| **Characteristic** | **No contrast**  N = 18^1^ | **Contrast**  N = 57^1^ | **p-value**^2^ | **q-value^3^** |
| --- | --- | --- | --- | --- |
| CFS |  |  | 0.2 | 0.8 |
| CFS 1-3 | 11 (61.11%) | 33 (57.89%) |  |  |
| CFS 4-6 | 2 (11.11%) | 17 (29.82%) |  |  |
| CFS 7-9 | 1 (5.56%) | 1 (1.75%) |  |  |
| Unknown | 4 (22.22%) | 6 (10.53%) |  |  |
| NELA score | 7 (3, 9) | 4 (1, 10) | 0.5 | 0.8 |
| Unknown | 10 | 32 |  |  |
| Operative approach |  |  | >0.9 | >0.9 |
| Laparoscopic | 4 (22.22%) | 14 (24.56%) |  |  |
| Laparoscopic converted to open | 3 (16.67%) | 10 (17.54%) |  |  |
| Open | 11 (61.11%) | 33 (57.89%) |  |  |
| Level of obstruction |  |  | 0.3 | 0.8 |
| Ileum | 9 (50.00%) | 31 (54.39%) |  |  |
| Jejunum | 3 (16.67%) | 3 (5.26%) |  |  |
| Unknown | 6 (33.33%) | 23 (40.35%) |  |  |
| Small bowel ischaemia description |  |  | 0.5 | 0.8 |
| Irreversible | 5 (27.78%) | 8 (14.04%) |  |  |
| Reversible | 1 (5.56%) | 5 (8.77%) |  |  |
| No ischaemia | 6 (33.33%) | 27 (47.37%) |  |  |
| Unknown | 6 (33.33%) | 17 (29.82%) |  |  |
| Small bowel resection | 7 (38.89) | 12 (21.05%) | 0.2 | 0.8 |
| ^1^n (%); Median (IQR) | | | | |
| ^2^Fisher's exact test; Wilcoxon rank sum test | | | | |
| ^3^False discovery rate correction for multiple testing | | | | |

**Supplementary Material 6: Outcomes of non-operative management, stratified by contrast type. (SBO = Small Bowel Obstruction, DVT = Deep Vein Thrombosis, PE = Pulmonary Embolism, HDU = High Dependency Unit, ITU = Intensive Therapy Unit).**

| **Characteristic** | **None**  N = 154^1^ | **Gastrografin**  N = 229^1^ | **Omnipaque**  N = 7^1^ | **Other**  N = 17^1^ | **p-value**^2^ | **q-value^3^** |
| --- | --- | --- | --- | --- | --- | --- |
| Length of stay (days) | 4 (2, 7) | 6 (3, 12) | 8 (3, 16) | 3 (2, 5) | **<0.001*** | **<0.001*** |
| Unknown | 1 | 1 | 0 | 0 |  |  |
| Time to surgery (days) | 1.0 (1.0, 2.0) | 3.0 (2.0, 5.8) | 2.0 (2.0, 2.0) | 3.0 (3.0, 3.0) | **<0.001*** | **0.001** |
| Unknown | 136 | 175 | 6 | 15 |  |  |
| Time to GI recovery (days) | 2.0 (1.0, 3.0) | 3.0 (2.0, 6.0) | 2.5 (1.3, 5.3) | 2.0 (1.0, 3.0) | **<0.001*** | **<0.001*** |
| Unknown | 25 | 25 | 1 | 1 |  |  |
| Operation for SBO | 18 (11.69%) | 54 (23.58%) | 1 (14.29%) | 2 (11.76%) | **0.021*** | 0.10 |
| Small bowel ischaemia description |  |  |  |  | 0.7 | 0.8 |
| No ischaemia | 6 (33.33%) | 25 (46.30%) | 0 (0.00%) | 2 (100.00%) |  |  |
| Reversible | 1 (5.56%) | 5 (9.26%) | 0 (0.00%) | 0 (0.00%) |  |  |
| Irreversible | 5 (27.78%) | 8 (14.81%) | 0 (0.00%) | 0 (0.00%) |  |  |
| Not stated | 6 (33.33%) | 16 (29.63%) | 1 (100.00%) | 0 (0.00%) |  |  |
| Unknown | 136 | 175 | 6 | 15 |  |  |
| Small bowel resection |  |  |  |  | >0.9 | >0.9 |
| Yes | 7 (4.55%) | 12 (5.24%) | 0 (0.00%) | 0 (0.00%) |  |  |
| No | 147 (95.45%) | 217 (94.76%) | 7 (100.00%) | 17 (100.00%) |  |  |
| In-hospital death | 9 (5.84%) | 12 (5.24%) | 0 (0.00%) | 1 (5.88%) | >0.9 | >0.9 |
| 30-day readmission | 27 (17.76%) | 23 (10.04%) | 0 (0.00%) | 2 (11.76%) | 0.13 | 0.3 |
| Unknown | 2 | 0 | 0 | 0 |  |  |
| Urinary tract infection | 4 (2.60%) | 7 (3.06%) | 1 (14.29%) | 0 (0.00%) | 0.4 | 0.5 |
| Pneumonia | 15 (9.74%) | 33 (14.41%) | 0 (0.00%) | 1 (5.88%) | 0.5 | 0.6 |
| Cardiac | 4 (2.60%) | 11 (4.80%) | 1 (14.29%) | 1 (5.88%) | 0.2 | 0.3 |
| DVT/PE | 2 (1.30%) | 3 (1.31%) | 1 (14.29%) | 0 (0.00%) | 0.2 | 0.3 |
| Delirium | 7 (4.55%) | 11 (4.80%) | 0 (0.00%) | 3 (17.65%) | 0.2 | 0.3 |
| Intra-abdominal sepsis | 4 (2.60%) | 5 (2.18%) | 0 (0.00%) | 1 (5.88%) | 0.6 | 0.7 |
| Radiological drainage | 0 (0.00%) | 1 (0.44%) | 0 (0.00%) | 1 (5.88%) | 0.11 | 0.3 |
| Unplanned HDU/ITU admission | 3 (1.95%) | 12 (5.24%) | 1 (14.29%) | 0 (0.00%) | 0.14 | 0.3 |
| Superficial surgical site infection | 0 (0.00%) | 2 (3.70%) | 0 (0.00%) | 1 (50.00%) | 0.13 | 0.3 |
| Unknown | 136 | 175 | 6 | 15 |  |  |
| Abdominal wall dehiscence | 0 (0.00%) | 1 (1.85%) | 0 (0.00%) | 0 (0.00%) | >0.9 | >0.9 |
| Unknown | 136 | 175 | 6 | 15 |  |  |
| Anastomotic leak |  |  |  |  |  |  |
| No | 18 (100.00%) | 54 (100.00%) | 1 (100.00%) | 2 (100.00%) |  |  |
| Unknown | 136 | 175 | 6 | 15 |  |  |
| Reoperation | 1 (5.56%) | 1 (1.85%) | 0 (0.00%) | 1 (50.00%) | 0.086 | 0.3 |
| Unknown | 136 | 175 | 6 | 15 |  |  |
| ^1^Median (IQR); n (%); Range | | | | | | |
| ^2^Kruskal-Wallis rank sum test; Fisher's exact test | | | | | | |
| ^3^False discovery rate correction for multiple testing | | | | | | |
| *Statistically significant | | | | | | |

**Supplementary Material 7: Comparison of operative outcomes in patients initially managed non-operatively with and without a contrast challenge. (DVT = Deep Vein Thrombosis, PE = Pulmonary Embolism, HDU = High Dependency Unit, ITU = Intensive Therapy Unit).**

| **Characteristic** | **No contrast**  N = 18^1^ | **Contrast**  N = 57^1^ | **p-value**^2^ | **q-value^3^** |
| --- | --- | --- | --- | --- |
| Time to GI recovery (days) | 4.0 (2.0, 5.0) | 3.5 (2.0, 5.3) | >0.9 | >0.9 |
| Unknown | 3 | 5 |  |  |
| Length of hospital stay (days) | 7 (6, 15) | 13 (9, 24) | **0.033*** | 0.4 |
| Unknown | 1 | 1 |  |  |
| In-hospital death | 2 (11.11%) | 2 (3.51%) | 0.2 | 0.8 |
| 30-day readmission | 2 (11.76%) | 3 (5.26%) | 0.3 | 0.8 |
| Unknown | 1 | 0 |  |  |
| Urinary tract infection | 0 (0.00%) | 3 (5.26%) | >0.9 | >0.9 |
| Pneumonia | 0 (0.00%) | 11 (19.30%) | 0.056 | 0.4 |
| Cardiac | 1 (5.56%) | 2 (3.51%) | 0.6 | 0.8 |
| DVT/PE | 1 (5.56%) | 2 (3.51%) | 0.6 | 0.8 |
| Delirium | 3 (16.67%) | 3 (5.26%) | 0.15 | 0.8 |
| Intra-abdominal sepsis | 2 (11.11%) | 5 (8.77%) | 0.7 | >0.9 |
| Radiological drainage | 0 (0.00%) | 2 (3.51%) | >0.9 | >0.9 |
| Unplanned HDU/ITU admission | 3 (16.67%) | 6 (10.53%) | 0.4 | 0.8 |
| Superficial surgical site infection (SSI) | 0 (0.00%) | 3 (5.26%) | >0.9 | >0.9 |
| Abdominal wall dehiscence | 0 (0.00%) | 1 (1.75%) | >0.9 | >0.9 |
| Anastomotic leak |  |  |  |  |
| No | 18 (100.00%) | 57 (100.00%) |  |  |
| Reoperation | 1 (5.56%) | 2 (3.51%) | 0.6 | 0.8 |
| ^1^n (%) | | | | |
| ^2^Pearson's Chi-squared test; Fisher's exact test | | | | |
| ^3^False discovery rate correction for multiple testing | | | | |
| *Statistically significant | | | | |

**Supplementary Material 8: Readmission data, stratified by contrast challenge administration and operative intervention following initial non-operative management. (CFS = Clinical Frailty Score. SBO = Small Bowel Obstruction. NOM = Non-Operative Management).**

|  | **Operated group** | | | **Non-operated group** | | |
| --- | --- | --- | --- | --- | --- | --- |
| **Contrast** | **No contrast**  N = 2^1^ | **Contrast**  N = 3^1^ | **p-value**^2^ | **No contrast**  N = 25^3^ | **Contrast**  N = 22^3^ | **p-value**^4^ |
| Age at time of admission (years) |  |  | >0.9 | 74 (59, 79) | 80 (73, 84) | **0.026*** |
| 33 | 0 (0%) | 1 (33%) |  |  |  |  |
| 39 | 1 (50%) | 0 (0%) |  |  |  |  |
| 46 | 1 (50%) | 0 (0%) |  |  |  |  |
| 69 | 0 (0%) | 1 (33%) |  |  |  |  |
| 73 | 0 (0%) | 1 (33%) |  |  |  |  |
| Sex |  |  | >0.9 |  |  | 0.5 |
| Female | 1 (50%) | 1 (33%) |  | 15 (60%) | 11 (50%) |  |
| Male | 1 (50%) | 2 (67%) |  | 10 (40%) | 11 (50%) |  |
| CFS |  |  | 0.4 |  |  | 0.4 |
| CFS 1-3 | 1 (50%) | 3 (100%) |  | 12 (48%) | 9 (41%) |  |
| CFS 4-6 | 0 (0%) | 0 (0%) |  | 10 (40%) | 7 (32%) |  |
| CFS 7-9 | 0 (0%) | 0 (0%) |  | 0 (0%) | 3 (14%) |  |
| Unknown | 1 (50%) | 0 (0%) |  | 3 (12%) | 3 (14%) |  |
| Reason for readmission |  |  | 0.2 |  |  | **0.032*** |
| Complication of surgery | 0 (0%) | 1 (33%) |  | NA (NA%) | NA (NA%) |  |
| Further episode of SBO | 2 (100%) | 0 (0%) |  | 19 (76%) | 10 (45%) |  |
| Other | 0 (0%) | 2 (67%) |  | 6 (24%) | 12 (55%) |  |
| Subsequent operation for index NOM admission | NA (NA%) | NA (NA%) |  | 4 (16%) | 0 (0%) | 0.11 |
| Unknown | 2 | 3 |  |  |  |  |
| ^1^n (%) | | | | | | |
| ^2^Fisher's exact test | | | | | | |
| ^3^Median (Q1, Q3); n (%) | | | | | | |
| ^4^Wilcoxon rank sum test; Pearson's Chi-squared test; Fisher's exact test | | | | | | |
| *Statistically significant | | | | | | |
